# Trends of relative and absolute socioeconomic equity in access to coronary revascularisations in 1995–2010 in Finland: a register study

**DOI:** 10.1186/s12939-017-0536-8

**Published:** 2017-02-20

**Authors:** Sonja Lumme, Kristiina Manderbacka, Ilmo Keskimäki

**Affiliations:** 1 Department of Health and Social Care Systems, Social and Health Systems Research Unit, National Institute for Health and Welfare, P.O. Box 30, FI-00271 Helsinki, Finland; 2 Faculty of Social Sciences, University of Tampere, Tampere, FI-33014 University of Tampere Finland

**Keywords:** Socioeconomic equity, Relative and absolute equity, Inequity indices, Coronary revascularisations, Register data, Need for care, IHD mortality

## Abstract

**Background:**

Resources for coronary revascularisations have increased substantially since the early 1990s in Finland. At the same time, ischaemic heart disease (IHD) mortality has decreased markedly. This study aims to examine how these changes have influenced trends in absolute and relative differences between socioeconomic groups in revascularisations and age group differences in them using IHD mortality as a proxy for need.

**Methods:**

Hospital Discharge Register data on revascularisations among Finns aged 45–84 in 1995–2010 were individually linked to population registers to obtain socio-demographic data. We measured absolute and relative income group differences in revascularisation and IHD mortality with slope index of inequality (SII) and concentration index (C), and relative equity taking need for care into account with horizontal inequity index (HII).

**Results:**

The supply of procedures doubled during the years. Socioeconomic distribution of revascularisations was in absolute and relative terms equal in 1995 (Men: SII = −12, C = −0.00; Women, SII = −30, C = −0.03), but differences favouring low-income groups emerged by 2010 (M: SII = −340, C = −0.08; W: SII = −195, C = −0.14). IHD mortality decreased markedly, but absolute and relative differences favouring the better-off existed throughout study years. Absolute differences decreased somewhat (M: SII = −760 in 1995, SII = −681 in 2010; W: SII = −318 in 1995, SII = −211 in 2010), but relative differences increased significantly (M: C = −0.14 in 1995, C = −0.26 in 2010; W: C = −0.15 in 1995, C = −0.25 in 2010). HII was greater than zero in each year indicating inequity favouring the better-off. HII increased from 0.15 to 0.18 among men and from 0.10 to 0.12 among women. We found significant and increasing age group differences in HII.

**Conclusions:**

Despite large increase in supply of revascularisations and decrease in IHD mortality, there is still marked socioeconomic inequity in revascularisations in Finland. However, since changes in absolute distributions of both supply and need for coronary care have favoured low-income groups, absolute inequity can be claimed to have decreased although it cannot be quantified numerically.

## Background

Studies from different countries have consistently reported poorer health and higher mortality among persons with lower socioeconomic position (e.g. [1]) including, e.g. increased ischaemic heart disease (IHD) incidence and mortality [2–4]. While IHD mortality has declined during the last decade in Finland and elsewhere [5], socioeconomic differences in it have increased [6]. A large part of the differences between socioeconomic groups derives from differences in common risk factors and health behaviours, but earlier studies have also reported socioeconomic differences in access to and quality of care. Among persons with IHD, similar differences have been reported in medicine use to prevent adverse cardiac events [7, 8], in access to investigations and invasive treatment [8–13], in use of cardiac rehabilitation [14, 15] and in outcomes of care [16, 17].

Some earlier studies suggest that an important factor behind socioeconomic differences in revascularisations may be supply of services. Earlier Finnish studies [18, 19] indicate that the overall level of revascularisations has a positive association with equity in the use of these services and that an increase in the overall level also increases equity. A similar positive association between procedure rates and socioeconomic equity has been reported in the UK [20] and in Sweden [21]. The overall level of revascularisations has more than doubled since the mid-1990s in Finland. This is especially true for percutaneous coronary intervention (PCI) while numbers of coronary artery bypass grafting (CABG) have decreased.

Need for treatment among IHD patients varies by several factors including age, gender, socioeconomic position, region and severity of disease [9, 22, 23]. Studies on socioeconomic equity in coronary revascularisation taking the need for care into account are scarce. Earlier studies have examined patients with acute myocardial infarction (AMI), (e.g. [12, 13, 24, 25]) and patients with unstable angina [21]. Assessing equity in revascularisations among all coronary heart disease patients is more complex; IHD mortality [11, 18–20], IHD incidence [11], and hospitalisations due to IHD [19] have been used as a proxy for need.

While IHD prevalence varies considerably by age, there are also age group differences in the treatment of IHD. The changing age structure of IHD patients, improved primary and secondary prevention and decreasing co-morbidity, growing life-expectancy, as well as improved medical technology have changed the treatment IHD [26, 27]. Despite these associations, only few studies have addressed age differences in socioeconomic equity in the treatment of IHD. Shaw et al. [25] have reported that older people in England in the 1990s probably received less revascularisations in relation to need. Manson-Siddle and Robinson [23] reported similar age differences in an ecological, area-based analysis from the former Yorkshire Region. Another study from the UK found no age differences in socioeconomic equality in treatment of AMI or secondary prevention of IHD by area-level socioeconomic position [28]. In Finland, Keskimäki et al. [19] found somewhat higher inequity favouring persons in higher social position among younger male patients compared to older male patients in access to coronary artery bypass grafting using IHD mortality as a proxy for need in the late 1980s. In this individual level register based study, the researchers used occupation (white/blue collar employees) as an indicator of social position. Among women the researchers report slightly greater inequity favouring white collar employees among the older patients. Another study from Finland using a similar setting (and social class position) detected higher inequity in revascularisations favouring white collar employees among younger patients among both genders in 1996 [18]. There is an obvious lack of recent studies addressing age differences in socioeconomic equity in access to coronary revascularisations using individual level data.

Inequalities are defined as differences in the presence of disease, health outcomes, or access to health care between socioeconomic groups. Inequities, on the other hand, are differences in health or health care that are not only unnecessary and avoidable but, in addition, are considered unfair and unjust [29, 30]. Earlier studies have pointed out that it is important to study both absolute and relative differences when examining equity or equality since presenting only one of these gives an accurate but inadequate picture of the differences and time trends in them [31]. For example, in many cases health or use of care may improve in all socioeconomic groups while the relative differences remain stable or increase [32, 33]. When studying equality in health or in health care without taking the need for care into account, both absolute and relative differences are easily measurable. However, studying equity in health care taking the need for care into account the situation is more complex. In studies where the need for care cannot be evaluated clinically in a case-by-case basis the variables of use of and need for care may be on different scales resulting in challenges in estimating absolute equity. In estimating relative equity, since relative disparity is scale invariant, the possible scale difference is not an issue since the variables are normalized and thus on the same scale. There are survey studies on absolute equity in health care taking the need into account in which need for care has been assessed as self-perceived health status or the presence of chronic disease (e.g. [34, 35]). However, self-reported health status is prone to report biases and the judgement of need for care is not clinical. Thus, it is not fully clear how it actually matches the clinical need for care. In register studies the need for care is usually evaluated using morbidity indicators as a proxy for need which are available at group-level (for example by gender, different age and socioeconomic groups) and thus does not describe the actual individual situation either. Additionally, in absolute differences a problem arises from the scale difference.

The aim of this paper was to develop approaches to extensively study trends in allocation of health care resources in relation to the need for care between socioeconomic groups using register data. We examined relative horizontal socioeconomic equity and also introduced a non-numerical approach to evaluate absolute horizontal socioeconomic equity in health care to fill the gap in the literature. We investigated a 16-year trend in socioeconomic equity in the use of coronary revascularisations taking the need for care into account, using IHD mortality as a proxy for need. During this time, the supply of coronary procedures increased while IHD mortality decreased substantially. In addition to age, gender, and socioeconomic position, we evaluated the need for care by also by region. We examined whether there are differences in socioeconomic equity in revascularisations between age groups. This study exploited comprehensive register data covering the whole population of Finland from 1995 to 2010 with all indicators measured at the individual level.

## Methods

### Study data

This study was based on register data on percutaneous coronary intervention (PCI) and coronary artery bypass grafting (CABG) among the non-institutionalized Finnish population aged 45–84 in 1995–2010. By means of the patients’ unique identification numbers, information on revascularisations obtained from the Finnish Hospital Discharge Register was linked to the population registries of Statistics Finland for data on socio-demographic factors including gender, age, income and region of residence.

We used disposable family income as an indicator for socioeconomic position. Family net income was adjusted for family size using the OECD modified equivalence scale [36]. The quintiles and fifth percentiles of the annual Finnish income distribution were used to categorise income into 5 and 20 groups. The same income limits were applied for men and women. We used university hospital districts, based on an administrative division of the Finnish hospital care system as an indicator of region of residence. The income record and hospital district for the year before the procedure was used. Age was grouped in five year age bands. Based on preliminary analyses, we also divided the data into two age groups, the younger (age groups 45–64) and the older (age groups 65–84) in some of the analyses to examine whether socioeconomic equity or socioeconomic differences in the distribution of revascularisations and IHD mortality varied by age.

The need of revascularisations was approximated using IHD mortality as a proxy for need. The number of IHD deaths of the resident population was obtained from the Causes of Death Register maintained by Statistics Finland. In all equity analyses gender, age and regional differences in the distributions of revascularisations and IHD mortality were taken into account.

We received data on the population at risk (i.e. person years) from Statistics Finland in tabulated form due to data protection regulations and tabulated the hospital data on revascularisations correspondingly. Thus analyses of the data were based on multidimensional tabulations of procedure data by gender, age, region of residence and income group.

### Statistical methods

To evaluate the annual crude and age-standardised rates of revascularisations and IHD mortality in 1995–2010, we calculated the numbers of revascularisations and IHD deaths in each age and gender group as a proportion of the person years of the corresponding population. All analyses were conducted separately for men and women. The crude age group rates (per 100 000 person years) were used to study differences and time-trends in the IHD mortality and the supply of revascularisations between age groups. We calculated directly age-standardised rates (per 100 000 person years) using the European population as the standard [37]. In the analyses of socioeconomic equity, we used annual age-standardised rates by 5 and 20 income groups. Income quintiles were used in the preliminary analyses and 20 income groups in the more detailed analyses of equity using inequity indices.

To measure relative socioeconomic differences in revascularisations and IHD deaths we used the concentration index (C), which summaries the magnitude of equality with one value, ranging from −1 to 1 and zero indicating equal distribution [38]. Negative values denote the concentration of the outcomes among the poorer and positive values concentration among the wealthier. The C is based on the concentration curve L(s), which enables the visualization of the distribution of the measured outcome variable. The concentration curve plots the cumulative proportion of the outcome variable against the cumulative proportion of the population (s) ranked by socioeconomic group (SEG) from the least to the most advantaged [39]. The area between the concentration curve and the diagonal provides a measure of inequality. If the L(s) lies below the diagonal, inequality exists favouring the less advantaged and vice versa. The C is defined as twice the area between the diagonal and the L(s) and can be computed numerically using a simple formula [38]. Kakwani [40] showed that the C can also be calculated using a weighted regression method as the estimate of *β*_1_, the slope of the regression line, in the following regression equation:

$$ \left[2{\sigma}_R^2\left(\frac{y_g}{y}\right)\right]{f}_g={\beta}_0{f}_g+{\beta}_1{R}_g{f}_g+{e}_g, $$

where *y*_*g*_ is the outcome variable measured on the *g* th SEG and *y* is the mean of the variable across all SEGs, and *f*_*g*_ is population share of the SEG. The *R*_*g*_ is the relative rank of the SEG and is defined as ${R}_g={\displaystyle {\sum}_{\gamma =1}^{g-1}}{f}_{\gamma}+0.5{f}_g$. The variance *σ*_*R*_^2^ is the weighted variance of the rank and defined as ${\sigma}_R^2={\displaystyle {\sum}_{g=1}^G}{f}_g{\left({R}_g-0.5\right)}^2.$

Absolute socioeconomic differences were measured using the slope index of inequality SII [41, 42]. The value of the SII can be interpreted as the difference in rates between the extremes of the income groups. Similarly to C, the negative values denote the concentration of the outcomes among the poorer (and vice versa). The greater the SII is, the greater the differences between the income groups are. It can be obtained by linear regression and the SII is the slope of the regression line:

$$ {y}_g{f}_g={\beta}_0{f}_g+{\beta}_1{R}_g{f}_g+{e}_{g.} $$

We used the horizontal inequity index (HII) as a measure of relative equity taking the need of care into account [43]. The HII is estimated by comparing the socioeconomic distribution of the supply of care (C_m_) to the socioeconomic distribution of the need of care C_n_. The values of the HII range from −2 to 2. We applied a statistical method to estimate the HII which allows taking into account the varying need for care in different socioeconomic and age groups in addition to regions [11]. This multilevel approach enabled us to study whether the varying levels of need between regions had an influence on the equity at the national level.

We estimated the confidence intervals for the C using the approach developed by Lumme et al. [44] and the confidence intervals for the HII and the SII were estimated developing further this approach. We used SAS (SAS Institute Inc., Cary, NC, USA) version 9.3 to analyse the data.

## Results

**Fig. 1:**
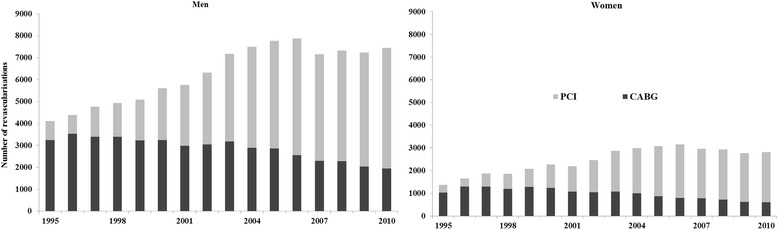
Number of revascularisations among the non-institutionalised Finnish population aged 45–84 in 1995–2010

According to the Finnish Hospital Discharge Register, the total number of performed coronary revascularisations was 4103 among men and 1373 among women in 1995 (Fig. 1). The overall number of procedures increased markedly until the mid-2000s, but the increase stabilised after that. By 2010, the supply had nearly doubled. In the beginning of the study period the majority of the performed procedures were CABGs. The share of PCIs grew throughout the period accounting for over 70% of all revascularisations among both genders in 2010.

**Fig. 2:**
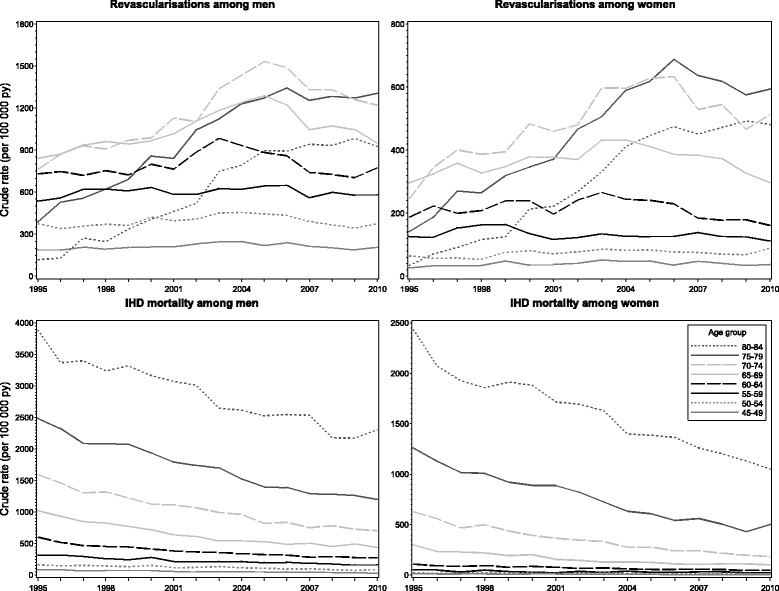
Crude revascularisation and ischaemic heart disease (IHD) mortality rates by age in 1995–2010 in Finland

Next we studied the change of crude revascularisation and IHD mortality rates (per 100 000 person years) by 5-year age bands from 1995 to 2010 (Fig. 2). Revascularisation rate remained at the same level among younger men (age groups 45–64) whereas the rate increased statistically significantly (*p*-values < 0.05) among older men (age groups 65–84). Among women, the revascularisation rate increased significantly in age group 50–54 and among those aged 70 years or older. IHD mortality rate decreased significantly in each age group among both genders throughout the study period.

In 1995, the age-standardised revascularisation rate (per 100 000 person years) was at the same level in the lowest and the highest income quintile (around 430 among men and around 140 among women). However, by 2010 the revascularisation rate increased significantly (*p*-values < 0.05) in the four lowest income quintiles but in the highest income quintiles the increase was modest and not statistically significant. Thus, in 2010 the rate was 1.5-fold higher among men and 1.9-fold among women in the lowest income quintile compared to the highest quintile. The age-standardised IHD mortality rate decreased evenly over the study period. In general, by 2010 the IHD mortality rate halved among men and was about 40% of the starting point level among women. The decreasing trend of IHD mortality rate was also significant (*p*-values < 0.05) in each income quintile among both genders. However, the rate ratio of the lowest and the highest income quintile increased from about 2 to 3.5 among both genders during the study period.

**Fig. 3:**
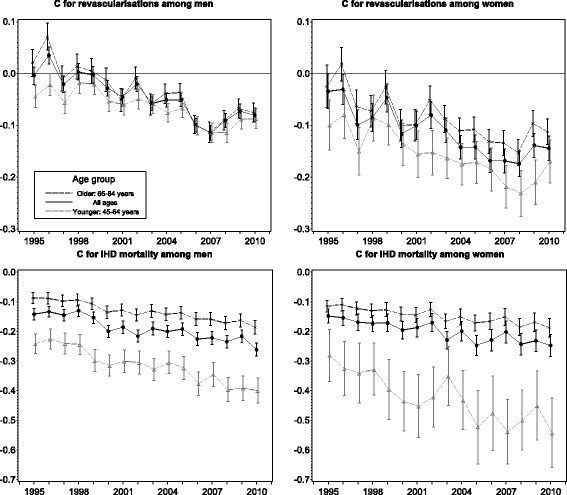
Concentration index (C) for revascularisations and ischaemic heart disease (IHD) mortality by age in 1995–2010 in Finland

The socioeconomic distribution of revascularisations as well as the distribution of IHD mortality in relative terms was estimated using the concentration index (C) (Fig. 3). In 1995, the C for revascularisations was −0.00 (95% confidence interval −0.02 to 0.01) among men indicating an equal distribution and −0.03 (95% CI −0.07 to 0.00) among women indicating minor differences favouring the low-income people. There was a clear decreasing trend (*p*-values < 0.0001) and by 2010 the C was as much as −0.08 (95% CI −0.09 to −0.07) among men and −0.14 (95% CI −0.17 to −0.12) among women. Due to different trends in the revascularisation rates between age groups, we studied the distribution of revascularisations also by age (younger: age groups 45–64 and older: age groups 65–84). In general, no significant difference in the distribution of revascularisations was found between these age groups among neither gender, with the exception of a few years. In 1995, the C for IHD mortality was −0.14 (95% CI −0.16 to −0.12) among men and −0.15 (95% CI −0.17 to −0.12) among women indicating evident differences with lower mortality among the rich (Fig. 3). Relative differences increased further (*p*-values for trend < 0.0001) over time and in 2010 the C was as high as −0.26 (95% CI −0.28 to −0.24) among men and −0.25 (95% CI −0.28 to −0.21) among women. The absolute value of the C was significantly higher in the younger age group throughout the study period among both genders. Furthermore, the difference between the age groups increased from 1995 to 2010, especially among women.

**Fig. 4:**
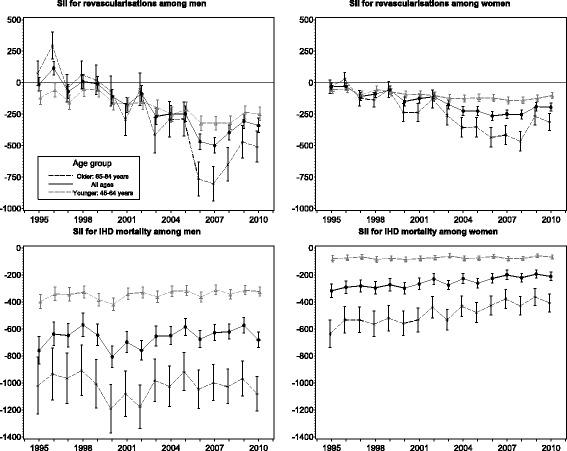
Slope index of inequality (SII) for revascularisations and ischaemic heart disease (IHD) mortality by age in 1995–2010 in Finland

In 1995, the value of the SII for revascularisations was around zero among both genders indicating equal distribution in revascularisations by income (Fig. 4). During the study period, the SII decreased significantly (*p*-values < 0.05), and in 2010 the SII  was −340 (95% CI −395 to −283) among men and −195 (95% CI −226 to −163) among women. In 1995 and 1996, the SII was positive among the older men and negative among the younger men, and these differences were significant. The absolute value of the SII was significantly higher from year 2006 onwards among the older men and from year 2003 onwards among the older women compared to the younger. In 1995, the SII for IHD mortality  was −760 (95% CI −860 to −657) among men and −318 (95% CI −368 to −266) among women indicating evident inequality favouring the rich. There was a slight improvement in absolute equality over time and in 2010 the SII was −681 (95% CI −738 to −623) among men and −211 (95% CI −241 to −179) among women. Among men the trend was not significant (*p* = 0.165), but among women it was significant (*p*-value < 0.0001). The absolute value of the SII was markedly greater among the older among both genders. A significant increasing trend was found only among older women, thus the difference between the age groups decreased among women.

**Fig. 5:**
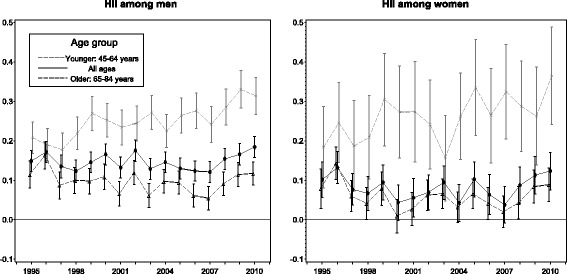
Horizontal inequity index (HII) for revascularisations need taken into account by age in 1995–2010 in Finland

Socioeconomic equity in revascularisations taking the need into account was estimated using the horizontal inequity index (HII). The need for care was estimated separately for each age and socioeconomic group in addition to regions. In 1995, the HII for the whole country was 0.15 (95% CI 0.12 to 0.18) among men and 0.10 (95% CI 0.06 to 0.15) among women indicating clear inequity favouring the rich (Fig. 5). Overall, the change was not significant during the study period. The HII was 0.18 (95% CI 0.16 to 0.21) among men in 2010. Among women the HII was at the same level in 2010 compared to year 1995, 0.12 (95% CI 0.08 to 0.17). The age group differences in HII were significant among men in 1995. Inequity increased significantly (*p*-values < 0.05) in the younger age groups resulting on marked differences in inequity between age groups among both genders in 2010.

## Discussion

This study examined socioeconomic equity in revascularisations in relation to need in Finland during a period of 16 years when the supply of revascularisations nearly doubled. Additionally, the proportion of PCI of all revascularisations increased significantly, being over 70% in 2010. However, the increase in revascularisation rates did not occur in all age groups: among men younger than 65 years and among women aged 45–49 and 55–69 years revascularisation rates did not increase during the study period. IHD mortality decreased in all age groups among both genders throughout the study period.

Our results showed that relative differences in revascularisations favouring low-income groups emerged among men and increased among women during the study period indicating improved access among the low-income groups. We did not detect differences between age groups in the socioeconomic distribution of revascularisations. In IHD mortality, however, income differences with lower rates among the better-off were found in the beginning of the study period and the differences enlarged significantly during the study period among both genders, with a more unequal distribution in younger age groups. In absolute terms, the income group distribution of revascularisations was equal in the beginning of the study period, but differences favouring the low-income groups emerged during the study period among both genders especially in older age groups. In IHD mortality the absolute differences with lower mortality among the better-off remained stable throughout the study period among men, but among women differences decreased. Additionally, the distribution was more unequal in the older age groups, but differences between age groups decreased markedly among women.

We found persistent and significant inequity in the use of revascularisation in relation to need favouring the better-off among both genders between 1995 and 2010. The need for care was evaluated separately also by regions in these analyses. Despite the increasing supply of revascularisations, the inequity did not decrease. In 1995, there were no age differences in equity among women, but inequity increased significantly among younger age groups during the study period resulting in significant age differences in equity among both genders.

There is a large body of research since the 1990s concerning socioeconomic differences in revascularisations [9, 12, 18, 19, 21–23, 25]. The results have generally been the same: the higher the socioeconomic position the larger the likelihood of revascularisation. There are some studies that have examined only persons with acute myocardial infarction [e.g. 13, 24] or acute coronary syndrome [45] or incident [10] or hospitalised [21] IHD patients thus having a more homogenous patient population. Additionally, IHD mortality [11, 18–20] and ischaemic heart disease incidence [11] and risk of hospitalisation due to IHD [18, 19] have been used as a proxy for need. However, most of these studies have not examined time-trends in revascularisations. There are only a few studies examining the effect of increasing supply of coronary care on socioeconomic equity taking the need into account. Manson-Siddle and Robinson [20] argue that increasing resources for tertiary cardiology without specific targeting may narrow inequity. Nevertheless, they recommend targeting of resources to the deprived. Hetemaa et al. [18] compared socioeconomic equity in revascularisations between 1988 and 1996 in Finland and conclude that despite substantial increase in coronary procedures, inequities diminished only somewhat. Haglund et al. [21] found diminishing socioeconomic inequalities between occupational groups with increasing resources and highlight the importance of identifying patients with the highest need of care. Contradictory to these studies, however, our results show persistent socioeconomic inequity by income groups despite increasing supply of revascularisations.

We found increasing inequity in the younger age groups while inequity remained at the same level in the older age groups between 1995 and 2010. Additionally, in 2010 inequity was significantly higher in the younger age groups. One possible explanation could be larger case-fatality among young coronary patients from lower income groups. We know of no studies examining socioeconomic differences in mortality before reaching the hospital by age. Earlier research suggests higher out-of-hospital and early (0–28 day) case-fatality among lower income group patients [4, 8] but register data from Finland suggest that the proportion of patients dying on day 0 is lower among younger age groups compared to older coronary patients [46]. It is therefore likely that inequity in revascularisations in relation to need found in the current study among younger age groups is also due to differences in access to treatment and not solely on higher out-of-hospital mortality.

It is a common finding that when mortality declines over time, absolute differences between socioeconomic groups tend to fall faster than relative differences. This finding was partly seen in our study as IHD mortality decreased in the last decades, but absolute differences remained stable in IHD mortality while relative differences increased. Despite a large increase in supply for revascularisations over time, we found marked socioeconomic inequity in revascularisations in relation to need in Finland. In relative terms, the evaluation of equity is straightforward. In absolute terms, this is more complex due to scale differences of the measures of use and need for care. The existing methods measuring absolute differences do not provide solutions for evaluating absolute inequity in health care taking the need for care into account. However, since in our study the changes of the absolute distributions of the both supply and the need for coronary care have favoured the low-income groups, our findings suggest that absolute inequity has decreased although it cannot be quantified numerically.

Register based studies usually do not provide direct indicators of the need for care, and thus we could not take into account clinical differences between patient groups. Nevertheless, IHD mortality, IHD incidence, and hospitalisations due to IHD are all group specific proxies for IHD morbidity and of these indicators mortality has most often been used as a proxy for the need for revascularisations in register studies using aggregated data. We used IHD mortality in this study as a proxy for the need which can be regarded as a limitation since it may overestimate socioeconomic differences in need. Earlier studies have reported worse survival after first MI and relatively more IHD deaths overall among low income patients [4, 47, 48]. However, using mortality as a proxy for need when total patient populations cannot be directly identified from the registers is feasible when using administrative register data, since the linkage of mortality information to the hospital registers is straightforward and mortality is simple to define. Moreover, IHD incidence might somewhat underestimate the true morbidity differences because the low income patients may be under-diagnosed and are diagnosed at a later stage when the disease is more severe. The indicator of region of residence we used in this study is rather broad; there are five university hospital districts in Finland. We were not able to use a more accurate indicator of region due to low numbers of events in some categories inducing unreliable estimates. However, the division of regions used in the study represents appropriately the notable differences in IHD mortality between eastern and western Finland.

The use of register data with long follow-up covering the whole population is one of the strengths of this study. The quality and coverage of the Finnish administrative registers are good [49, 50]. Additionally, the validity of diagnoses of major coronary events in the Causes of Death Register is good [51, 52]. The use of the concentration index as a measure of equity is a methodological strength in our study; the C summarises information considerably and thus enables taking into account several dimensions simultaneously.

## Conclusions

Despite a large increase in resources for coronary interventions in the last decades, there is still marked relative inequity between socioeconomic groups in access to revascularisation in relation to need in Finland. More effective measures are needed to secure equity in coronary care. It seems that untargeted increase in resources may not be sufficient to further decrease differences in access to operations. Instead, identifying patients with the highest need of care early and more specific targeting of resources especially to middle-aged low income coronary patients is needed, since the improvement of cardiovascular health among this patient group has been slower.

## Abbreviations

AMI: Acute myocardial infarction
C: Concentration index
CABG: Coronary artery bypass grafting
HII: Horizontal inequity index
IHD: Ischaemic heart disease
PCI: Percutaneous coronary intervention
SII: Slope index of inequality
